# Supplementary material for: Structure of the human Bre1 complex bound to the nucleosome
Source: Nat Commun. 2024 Mar 22;15:2580. doi: 10.1038/s41467-024-46910-8 (PMC10959955; doi:10.1038/s41467-024-46910-8)
Supplement: Supplementary file 1 — Supplementary Information [file 41467_2024_46910_MOESM1_ESM.pdf]

Supplementary Information

Structure of the human Bre1 complex bound to the nucleosome

Shuhei Onishi[1], Kotone Uchiyama[1], Ko Sato[1], Chikako Okada[1], Shunsuke Kobayashi[1], Keisuke Hamada[1], Tomohiro Nishizawa[2,3], Osamu Nureki[2], Kazuhiro Ogata[1,*], and Toru Sengoku[1,*]

[1]Department of Biochemistry, Yokohama City University Graduate School of Medicine, Yokohama, Japan
[2]Department of Biological Sciences, Graduate School of Science, The University of Tokyo, Tokyo, Japan
[3]Present address: Graduate School of Medical Life Science, Yokohama City University, Yokohama, Japan
*To whom correspondence should be addressed:
ogata@yokohama-cu.ac.jp and tsengoku@yokohama-cu.ac.jp

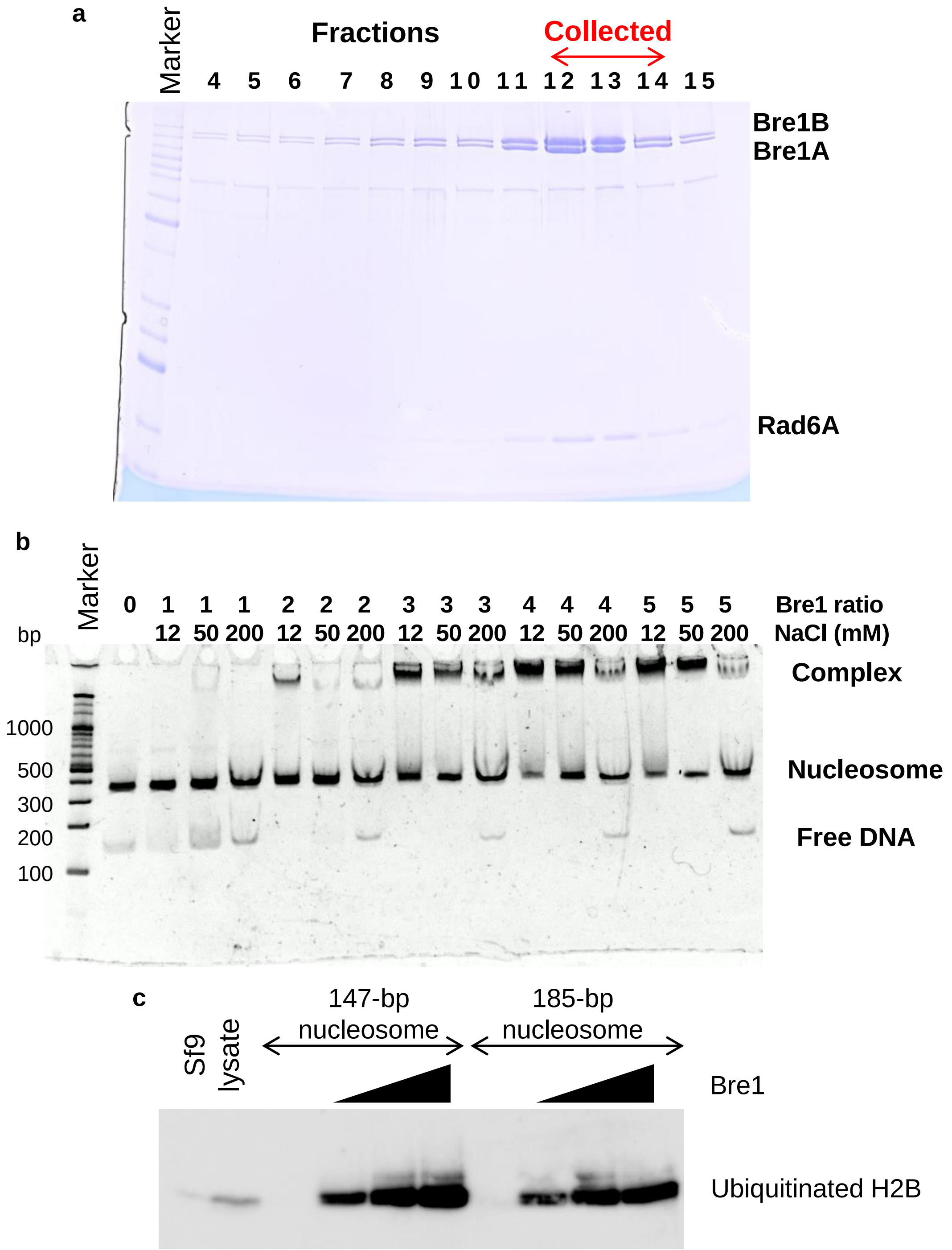

Supplementary Figure 1: Purification and characterization of the human Bre1 complex. a, Purification of the trimeric Bre1A-Bre1B-Rad6A complex using a Superose 6 column. Fractions 12–14 were pooled and used for further study. **b**, Interaction between the trimeric Bre1 complex and the nucleosome with 147-bp DNA analyzed using non-denaturing gel electrophoresis under different Bre1-to-nucleosome molar ratios and NaCl concentrations. **c**, Ubiquitination of nucleosomal H2BK120 by the trimeric Bre1 complex (at 0.5, 1.0, or 1.5 μM) analyzed using western blotting. Sf9 lysate was also loaded as a control. Source data are provided as a Source Data file.

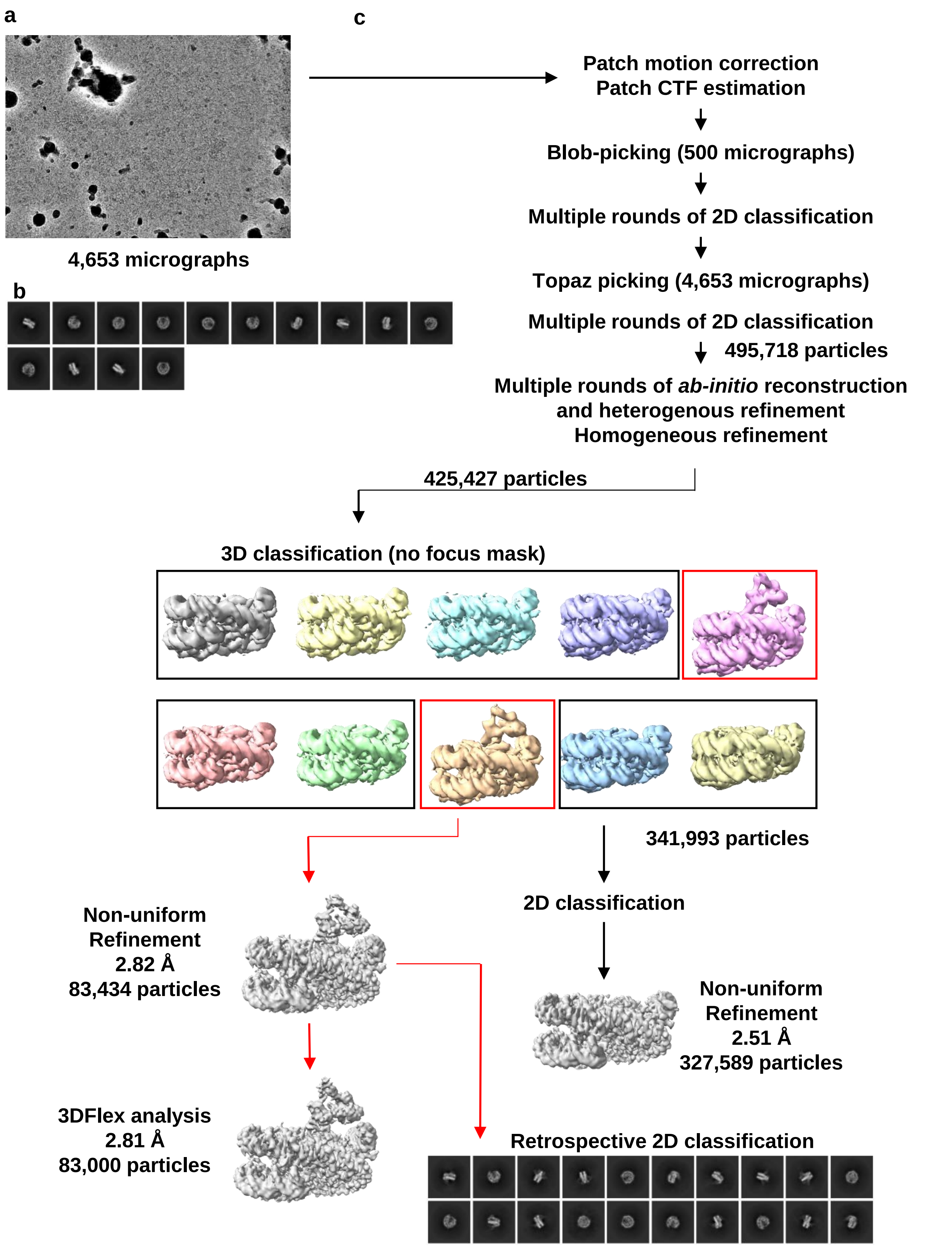

Supplementary Figure 2: Cryo-EM processing workflow. a, Representative motion-corrected micrograph. **b**, Two-dimensional class averages. **c**, Flowchart of data processing. The result of the retrospective 2D classification shows additional density for the bound Bre1 complex.

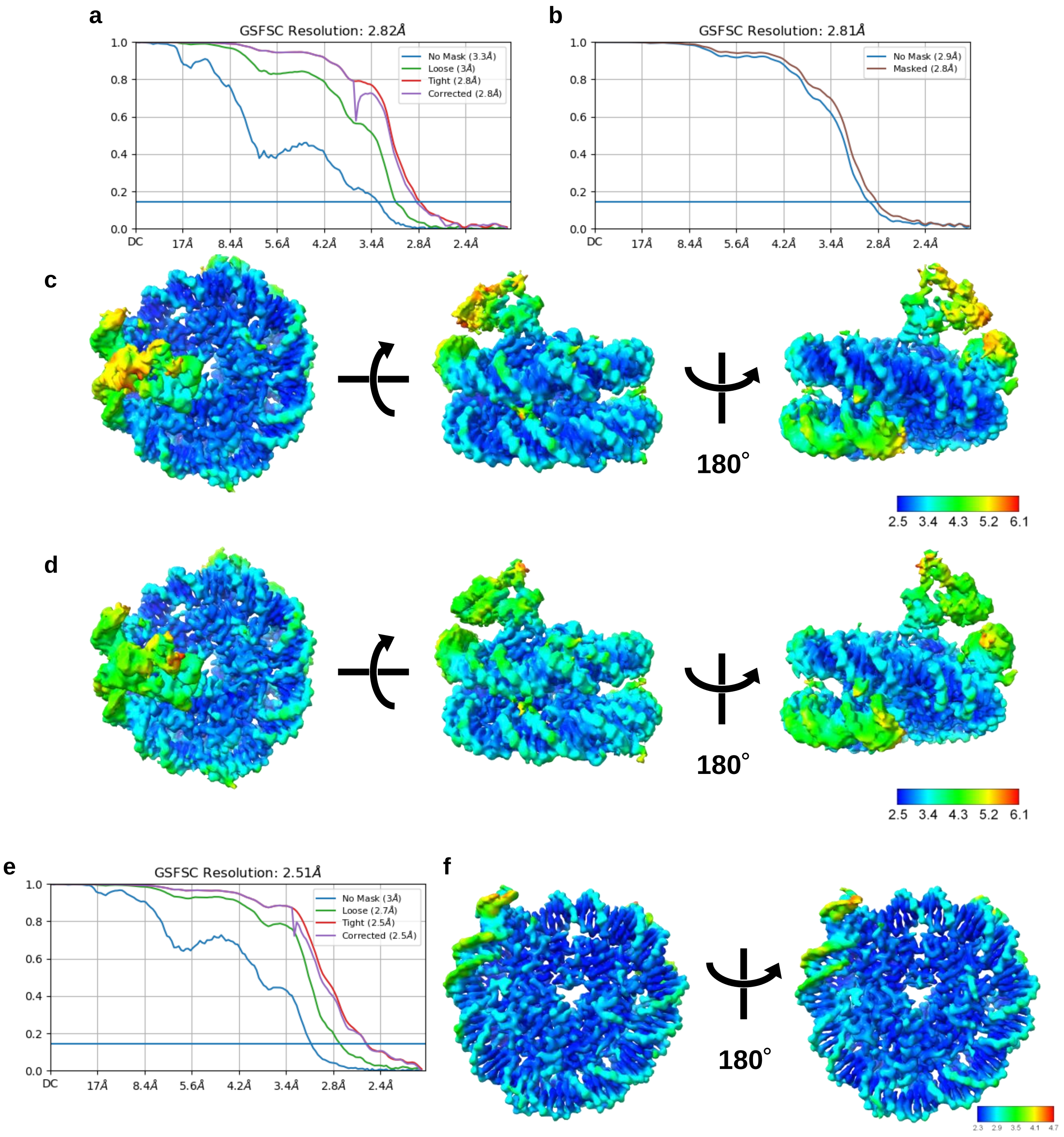

Supplementary Figure 3: Validation of the cryo-EM maps. a and b, FSC curve for the Bre1-nucleosome density map after Non-uniform refinement (a) and after 3DFlex analysis (b). **c and d,** Local resolution map of Bre1-nucleosome after Non-uniform refinement (c) and after 3DFlex analysis (d). **e**, FSC curve for the free nucleosome density map after Non-uniform refinement. **f,** Local resolution map of the free nucleosome after Non-uniform refinement.

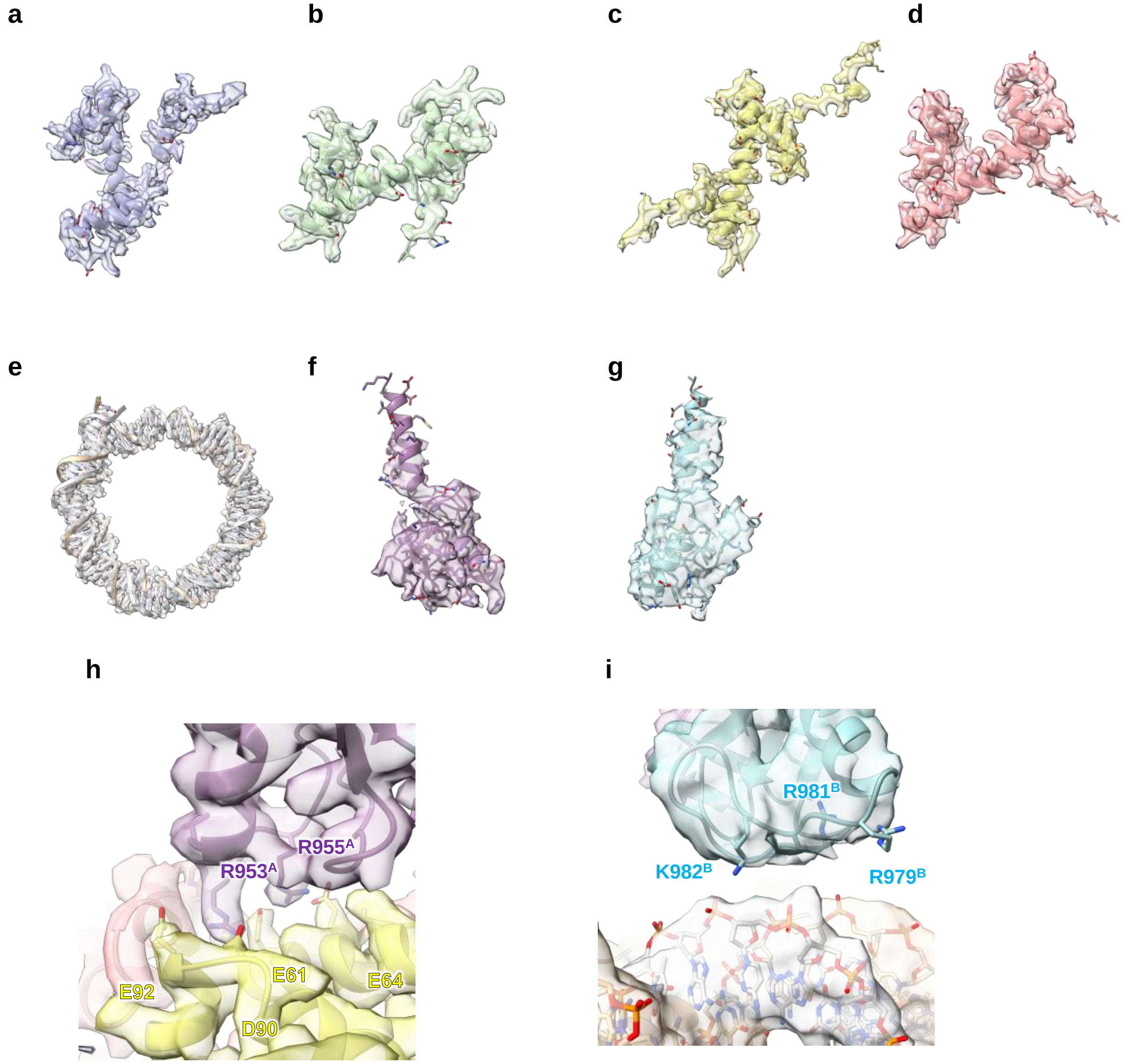

Supplementary Figure 4: Density map of the Bre1-nucleosome complex. a, H3. **b**, H4. **c**, H2A. **d**, H2B. **e**, DNA. **f**, RING domain bound to the acidic patch (modeled here as $RING^A$). **g**, RING domain bound to the DNA phosphates (modeled here as $RING^B$). **h**, Close-up view near the arginine anchor. **i**, Close-up view of the $RING^B$-DNA interface.

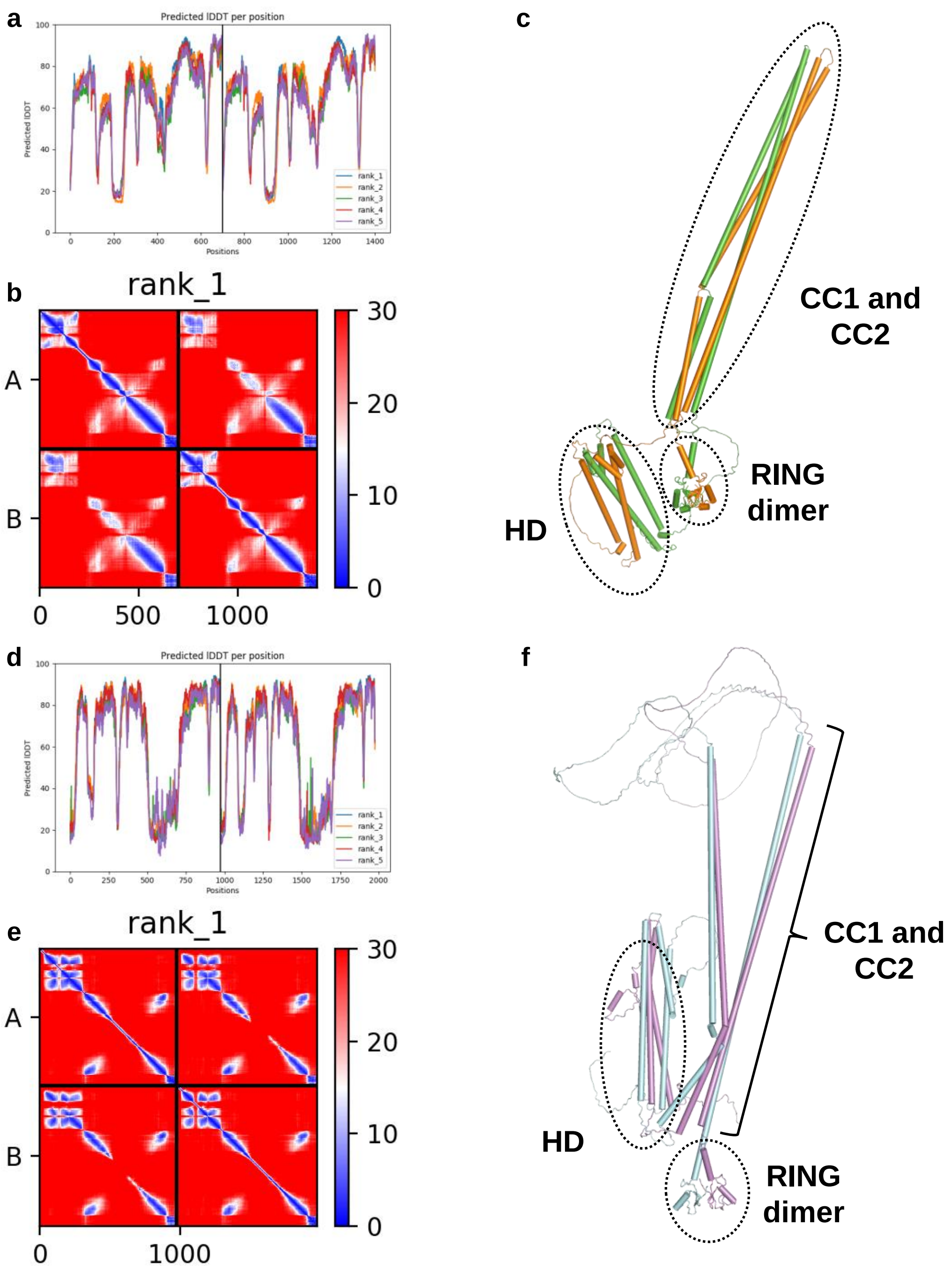

Supplementary Figure 5: Model structures of yeast (a-c) and human (d-f) Bre1 complexes calculated using AlphaFold. a, d, Distribution of the predicted local distance difference test (lDDT) values of five model structures. **b, e**, Distribution of the predicted aligned error (PAE) values (in Å) of the top-ranked model. **c, f**, Overall structure of the top-ranked models. The two yeast Bre1 molecules are colored green and orange.

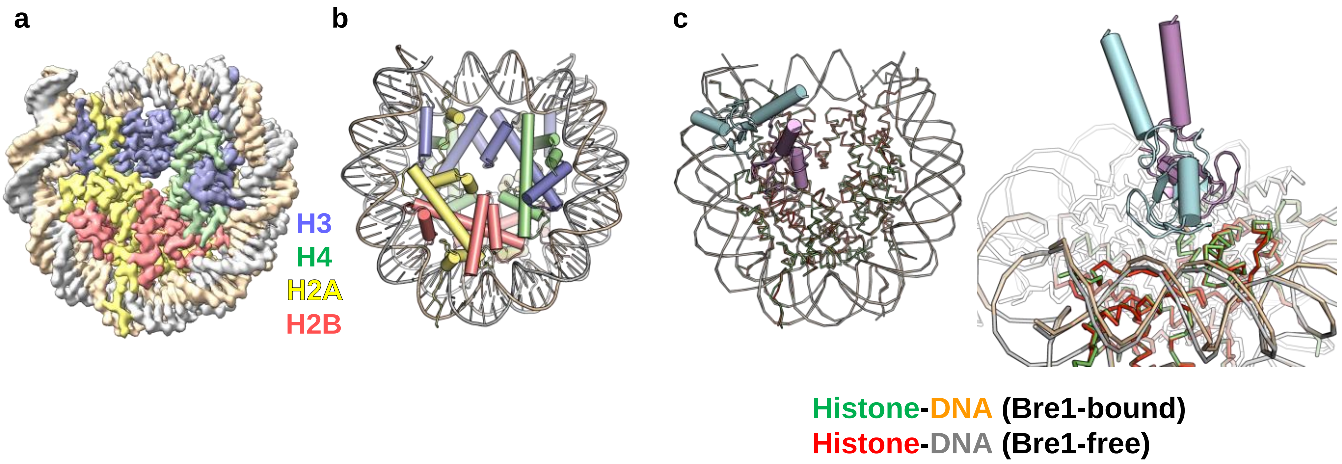

Supplementary Figure 6: Small conformational change of the nucleosomal DNA observed on Bre1 binding. a, Density map of the Bre1-free nucleosome at 2.51 Å resolution. **b**, Cartoon representation. **c**, Superposition of the Bre1-bound and Bre1-free nucleosome structures determined in this study (overall and closeup views). $RING^{A}$ and $RING^{B}$ are shown as a cartoon model, whereas histones and DNA are shown as a backbone trace model. Histones and DNA are colored green and orange in the Bre1-bound nucleosomes, and red and gray in the Bre1-free nucleosomes.

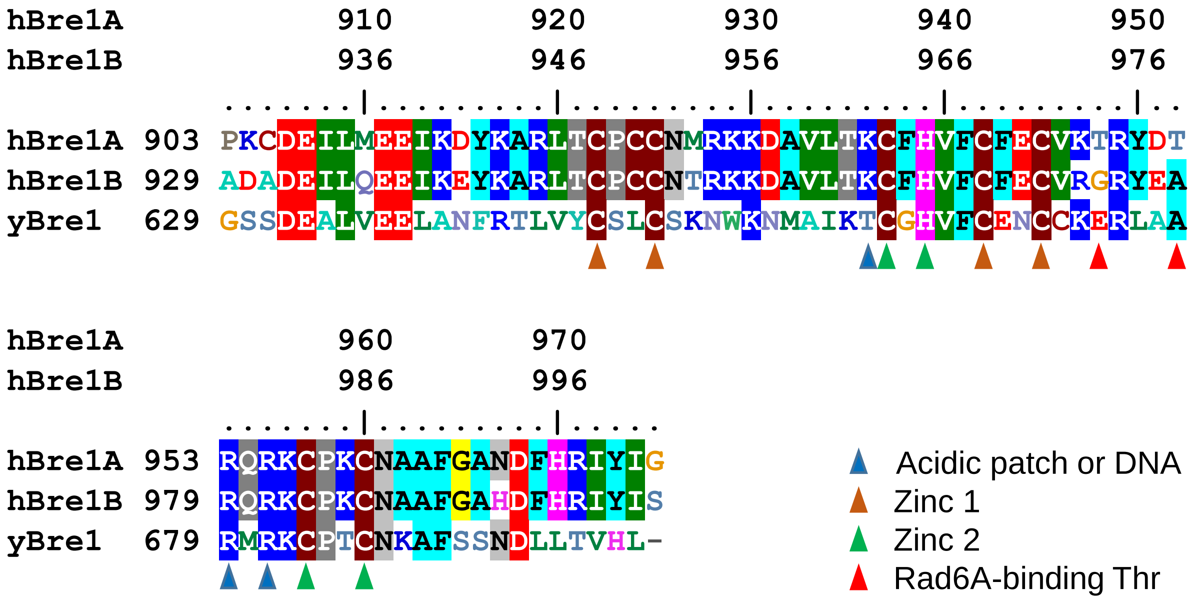

Supplementary Figure 7: Sequence alignment of the RING domains of yeast Bre1, human Bre1A, and human Bre1B. Residues that contact the acidic patch, DNA phosphates, or zinc ions are indicated. The two threonine residues of Bre1A (T948^{A} and T952^{A}) that are possibly involved in Rad6A binding are also indicated.

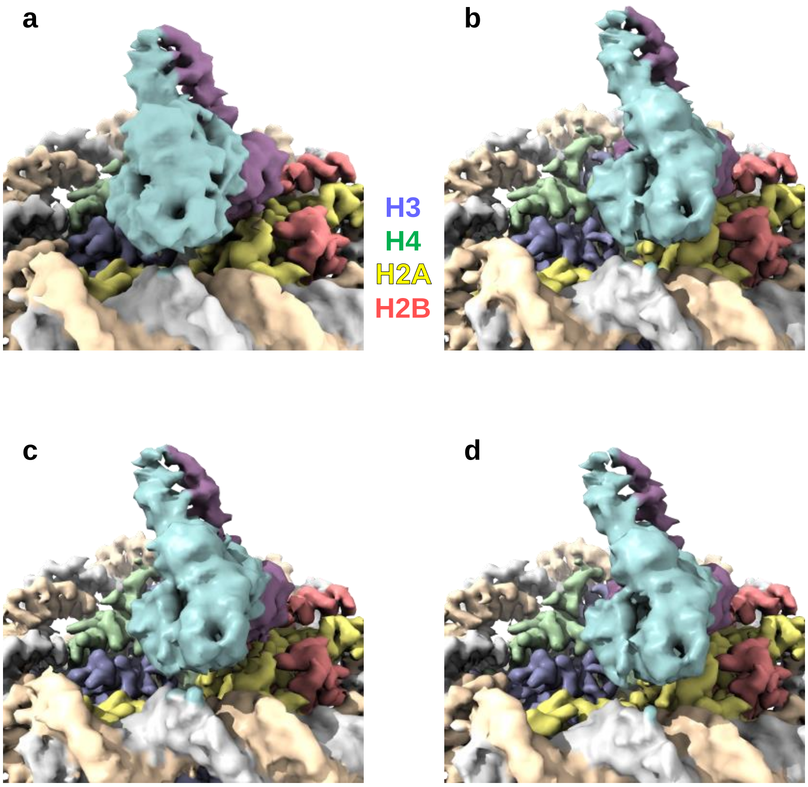

Supplementary Figure 8: Convected density maps. Convected density maps corresponding to the latent coordinates on either side of the dimension 0 axis (**a** and **b**) and the dimension 1 axis (**c** and **d**).

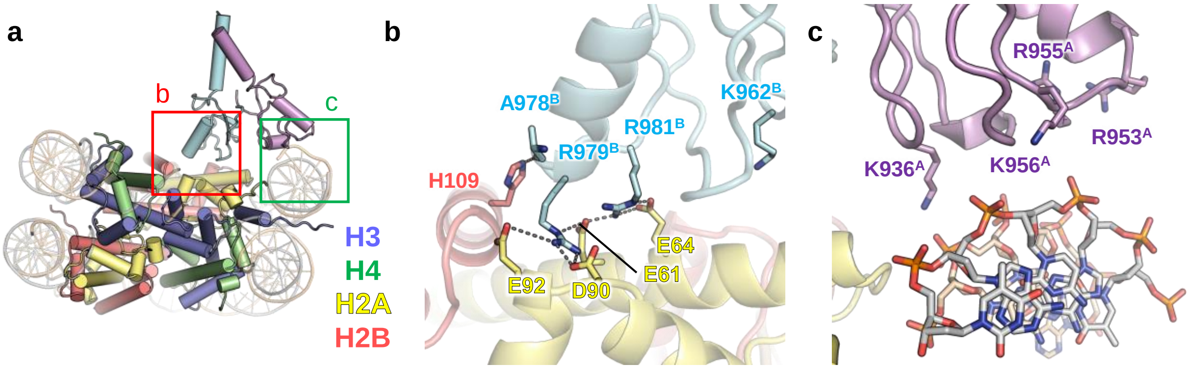

Supplementary Figure 9: Interactions between the Bre1 complex and the nucleosome in Model II. a, Structure of Model II. The magnified regions in b and c are indicated. **b**, Interactions between RING^B and the acidic patch. Hydrophilic interactions (salt bridges and hydrogen bonds) are indicated by gray dotted lines. **c**, Basic residues of RING^A near the DNA phosphates.

a

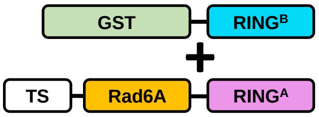

b

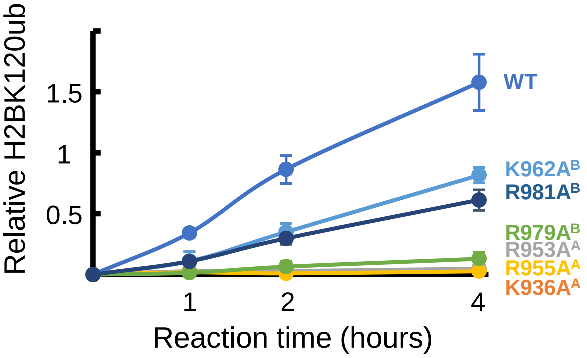

Supplementary Figure 10: H2BK120 ubiquitination assay using GST-tagged RING^B and Twin-Strep-tagged Rad6A-RING^A fusion. a, A schematic representation of the protein constructs used in the experiment shown in b. **b**, H2BK120 ubiquitination assay of the wild type (WT) and mutants possessing substitutions at basic residues. The signals were normalized using the normalization control. The mean and standard deviation of three independent results are shown. Source data are provided as a Source Data file.

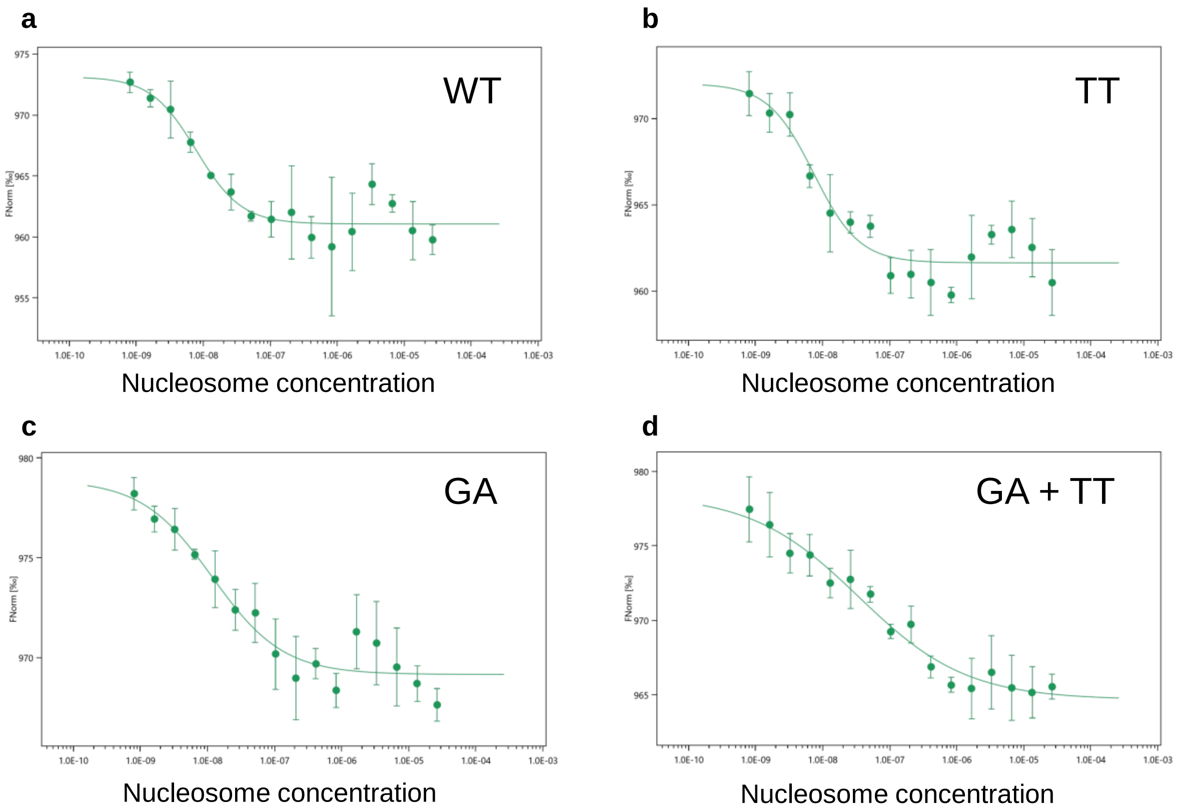

Supplementary Figure 11: Microscale thermophoresis analysis of interactions between Bre1 complexes and the nucleosome. a, WT. **b**, the TT mutant. **c**, the GA mutant. **d**, the GA + TT mutant.

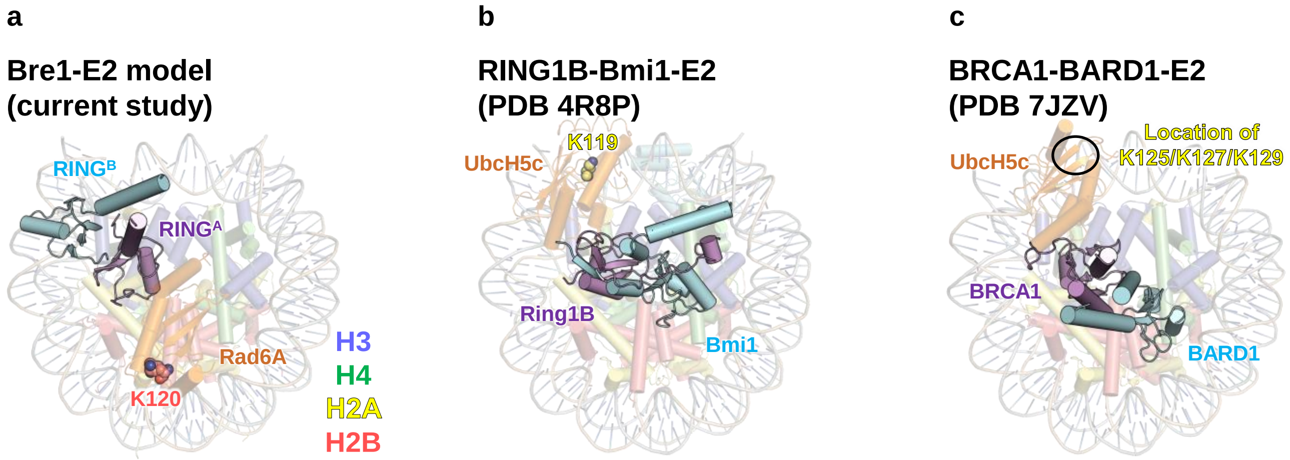

Supplementary Figure 12: Comparison of nucleosomal histone ubiquitin ligases. a, Model structure of RING^{A}-RING^{B}-Rad6A-ubiquitin bound with the nucleosome (ubiquitin omitted for clarity). **b**, Structure of the RING1B-Bmi1 complex and its E2 enzyme, UbcH5c, bound to the nucleosome. **c**, Structure of the BRCA1-BARD1 complex and its E2 enzyme, UbcH5c, bound to the nucleosome. The target residues (H2AK125, K127, and K129) are disordered in the structure.
