# Supplementary material for: Structure of the human Bre1 complex bound to the nucleosome
Source: Nat Commun. 2024 Mar 22;15:2580. doi: 10.1038/s41467-024-46910-8 (PMC10959955; doi:10.1038/s41467-024-46910-8)
Supplement: Supplementary file 3 — Description of Additional Supplementary Files [file 41467_2024_46910_MOESM3_ESM.pdf]

Description of Additional Supplementary Files

File Name: Supplementary Data 1
Description: CRISPR knockdown data for Bre1A (RNF20) and Bre1B (RNF40) downloaded from Depmap.

File Name: Supplementary Data 2
Description: Sequences of recombinant proteins and DNA.

File Name: Supplementary Movie 1
Description: Density map and overall structure of Model I.

File Name: Supplementary Movie 2
Description: A series of the convected densities along the first dimension.

File Name: Supplementary Movie 3
Description: A series of the convected densities along the second dimension.

File Name: Supplementary Movie 4
Description: Interactions between arginine anchors and the acidic patch.

File Name: Supplementary Movie 5
Description: A model structure of the RINGA-RINGB-Rad6A-ubiquitin complex bound to the nucleosome.
