# Supplementary material for: Structure of the human Bre1 complex bound to the nucleosome
Source: Nat Commun. 2024 Mar 22;15:2580. doi: 10.1038/s41467-024-46910-8 (PMC10959955; doi:10.1038/s41467-024-46910-8)
Supplement: Supplementary file 11 — Reporting Summary [file 41467_2024_46910_MOESM11_ESM.pdf]

nature portfolio

Corresponding author(s): Toru Sengoku

Last updated by author(s): Feb 16, 2024

Reporting Summary

Nature Portfolio wishes to improve the reproducibility of the work that we publish. This form provides structure for consistency and transparency in reporting. For further information on Nature Portfolio policies, see our Editorial Policies and the Editorial Policy Checklist.

Statistics

For all statistical analyses, confirm that the following items are present in the figure legend, table legend, main text, or Methods section.

n/a	Confirmed	
☐	☒	The exact sample size (*n*) for each experimental group/condition, given as a discrete number and unit of measurement
☐	☒	A statement on whether measurements were taken from distinct samples or whether the same sample was measured repeatedly
☒	☐	The statistical test(s) used AND whether they are one- or two-sided<br>*Only common tests should be described solely by name; describe more complex techniques in the Methods section.*
☒	☐	A description of all covariates tested
☒	☐	A description of any assumptions or corrections, such as tests of normality and adjustment for multiple comparisons
☐	☒	A full description of the statistical parameters including central tendency (e.g. means) or other basic estimates (e.g. regression coefficient) AND variation (e.g. standard deviation) or associated estimates of uncertainty (e.g. confidence intervals)
☒	☐	For null hypothesis testing, the test statistic (e.g. *F*, *t*, *r*) with confidence intervals, effect sizes, degrees of freedom and *P* value noted<br>*Give P values as exact values whenever suitable.*
☒	☐	For Bayesian analysis, information on the choice of priors and Markov chain Monte Carlo settings
☒	☐	For hierarchical and complex designs, identification of the appropriate level for tests and full reporting of outcomes
☒	☐	Estimates of effect sizes (e.g. Cohen's *d*, Pearson's *r*), indicating how they were calculated

Our web collection on statistics for biologists contains articles on many of the points above.

Software and code

Policy information about availability of computer code

Data collection	SerialEM v3.7.4, Compass for SW v6.1.0
Data analysis	AlphaFold v2.3.2, LocalColabFold v1.5.0, CryoSPARC v4.2.1,Topaz v0.2.5, Coot v0.9.8.7, PyMOL v2.5.0, ChimeraX v1.4, Compass for SW v6.2.0, MO. Affinity Analysis v2.3

For manuscripts utilizing custom algorithms or software that are central to the research but not yet described in published literature, software must be made available to editors and reviewers. We strongly encourage code deposition in a community repository (e.g. GitHub). See the Nature Portfolio guidelines for submitting code & software for further information.

Data

Policy information about availability of data

All manuscripts must include a data availability statement. This statement should provide the following information, where applicable:

- Accession codes, unique identifiers, or web links for publicly available datasets
- A description of any restrictions on data availability
- For clinical datasets or third party data, please ensure that the statement adheres to our policy

The cryo-EM density maps and models were deposited in the Electron Microscopy Data Bank (EMD-34274 and EMD-34275) and Protein Data Bank (8GUI, 8GUJ, and 8GUK).

Research involving human participants, their data, or biological material

Policy information about studies with human participants or human data. See also policy information about sex, gender (identity/presentation), and sexual orientation and race, ethnicity and racism.

Reporting on sex and gender	No concern about this section in this study.
Reporting on race, ethnicity, or other socially relevant groupings	No concern about this section in this study.
Population characteristics	No concern about this section in this study.
Recruitment	No concern about this section in this study.
Ethics oversight	No concern about this section in this study.

Note that full information on the approval of the study protocol must also be provided in the manuscript.

Field-specific reporting

Please select the one below that is the best fit for your research. If you are not sure, read the appropriate sections before making your selection.

☒ Life sciences ☐ Behavioural & social sciences ☐ Ecological, evolutionary & environmental sciences

For a reference copy of the document with all sections, see nature.com/documents/nr-reporting-summary-flat.pdf

Life sciences study design

All studies must disclose on these points even when the disclosure is negative.

Sample size	No statistical methods were used to determine the sample size.
Data exclusions	No data was excluded in this study.
Replication	Three to six independent experiments were performed in enzymatic and binding assays and all replication attempts were successful. Protein expression and purification from insect cells was successfully repeated more than three times. No attempt was made to replicate the other experiments.
Randomization	For cryo-EM analysis, particles were randomly assigned to half-maps for resolution estimation. Randomization was not used in the other experiments as it was not relevant to them.
Blinding	Blinding was not relevant to the experiments in our study since no subjective allocation was involved in this study.

Reporting for specific materials, systems and methods

We require information from authors about some types of materials, experimental systems and methods used in many studies. Here, indicate whether each material, system or method listed is relevant to your study. If you are not sure if a list item applies to your research, read the appropriate section before selecting a response.

Materials & experimental systems

n/a	Involved in the study	
☐	☒	Antibodies
☒	☐	Eukaryotic cell lines
☒	☐	Palaeontology and archaeology
☒	☐	Animals and other organisms
☒	☐	Clinical data
☒	☐	Dual use research of concern
☒	☐	Plants

Methods

n/a	Involved in the study	
☒	☐	ChIP-seq
☒	☐	Flow cytometry
☒	☐	MRI-based neuroimaging

Antibodies

Antibodies used	Ubiquityl-Histone H2B (Lys120) (D11) Rabbit mAb (No. 5546; Cell Signaling), diluted 1:1000 for Western blotting and 1:250 for Wes analysis.<br>goat anti-rabbit IgG-HRP (sc-2004; Santa Cruz Biotechnology), diluted 1:2000 for Western blotting.<br>Anti-Rabbit Detection Module (DM-001; ProteinSimple), used without dilution.
Validation	The antibodies was validated by the manufacture (https://www.cellsignal.jp/products/primary-antibodies/ubiquityl-histone-h2b-lys120-d11-xp-rabbit-mab/5546, https://www.scbt.com/ja/p/goat-anti-rabbit-igg-hrp, https://www.bio-techne.com/p/simple-western/anti-rabbit-detection-module_dm-001).

Plants

Seed stocks	No plant experiment was performed in this study.
Novel plant genotypes	No plant experiment was performed in this study.
Authentication	No plant experiment was performed in this study.
